# Education differences in cardiometabolic risk in England, Scotland and the United States between 1992 and 2019

**DOI:** 10.1186/s12872-022-02681-y

**Published:** 2022-06-02

**Authors:** Diego Montano

**Affiliations:** Department of Population-Based Medicine, Institute for Health Sciences, University of Tübingen, Hoppe-Selyer-Str. 9, 72076 Tübingen, Germany

**Keywords:** Epidemiology of cardiovascular disease, Cardiovascular risk scores, Health survey data, Social determinants of health

## Abstract

**Background:**

Education differences in cardiometabolic risk and disease still play a major role in the magnitude of the socioeconomic health disparities in high-income societies. However, the knowledge on how education differences may have changed over time regarding the distribution of multiple risk factors is rather limited. This study aims to provide a comprehensive assessment of the magnitude of those differences in three high-income countries.

**Methods:**

Data from repeated cross-sectional population health and examination surveys conducted between 1992 and 2019 in England, Scotland and the United States are analysed (pooled sample size $n = 362{,}275$). Six cardiometabolic risk factors, namely, systolic and diastolic blood pressure, body-mass-index, glycated haemoglobin HbA1c, serum total cholesterol and the cardiovascular risk score are analysed with linear mixed models.

**Results:**

Education differences in cardiometabolic risk were found to have either increased or remained stable for the past 3 decades in the countries included in the analyses. Among individuals with no qualification the cardiometabolic risk has been higher than among the higher educated (mean difference: 0.136, 99% CI [0.119; 0.152]). Education differences were observed also for systolic blood pressure (2.788 mmHg, 99% CI [2.529; 3.047]), glycated haemoglobin HbA1c (0.160 %, 99% CI [0.136; 0.185]), total cholesterol (0.268 mmol/L, 99% CI [0.247; 0.289]) and body-mass-index (0.591 kg/m^2^, 99% CI [0.504; 0.679]).

**Conclusion:**

The results suggest a more complex pattern of associations between education and health which may be due to education-dependent processes related to behavioural, cognitive and attitudinal modification and adaptation to changing socio-cultural conditions.

**Supplementary Information:**

The online version contains supplementary material available at 10.1186/s12872-022-02681-y.

## Background

In the past 2 decades, several epidemiological studies have provided important results concerning the temporal trends in the distribution of various cardiometabolic risk factors and cardiovascular disease (CVD) at the population level. Previous findings indicate that these trends may include both increase or decrease of cardiometabolic risk over time, depending on the type of risk factor under consideration. For instance, in recent decades several trends have been observed in Europe and other high income countries: (i) a decrease of average systolic and diastolic blood pressure (BP) [1], (ii) an increase in average body-mass-index (BMI) measurements [2], (iii) a relatively constant prevalence rate of diabetes mellitus [3], (iv) a higher prevalence rate of insufficient physical activity [4] and (v) a lower incidence of fatal and non-fatal heart attacks [5, 6].

At the same time, however, it has been found that the socioeconomic status of individuals still accounts for a substantial proportion of observed variation in cardiometabolic risk and CVD outcomes; in particular, the associations of the individual’s educational attainment with several cardiometabolic health outcomes have been large and consistent [7–13]. Some previous research findings have suggested that education plays a more important role than income in explaining the health differences observed for indicators of the socioeconomic status [7, 14, 15]. Some instances of such educational disparities regarding the cardiometabolic risk include higher cardiovascular and overall mortality risk in Europe [7, 8] and higher frequency of metabolic syndrome, myocardial infarction or sudden cardiac death among persons with lower educational attainment [9, 10]. Moreover, despite the fact that the average number of years of life lost has been decreasing in several European countries, recent analyses have suggested that this decrease has been more pronounced among individuals with higher education than among the low educated [11].

Nonetheless, one of the limitations of previous epidemiological research on population-based time trends and education differences in cardiometabolic health outcomes is the fact that the analyses have been frequently performed with univariate time series (e.g., only blood pressure) or aggregated national data (e.g., national health statistics) [1, 8, 11]. As a consequence, the inherent complexity associated with the distribution of the cardiometabolic risk in the population has not been addressed adequately in terms of phenomena such as the co-variation of single risk factors, the temporal changes in the age and sex distribution of the population or the extent to which the distribution of several cardiometabolic risk factors varies across educational levels. Hence, the knowledge on how education differences in cardiometabolic risk may have changed over time regarding the distribution of multiple cardiometabolic risk factors is rather limited. This research gap contrasts with the indications of previous research suggesting that education differences in cardiometabolic health outcomes should not be expected to decrease, even if the educational level of the population as a whole increases [16–18]. It has been suggested that the health benefits attributable to education would result not from the educational certificates *per se*, but from the expansion of cognitive resources which ensue from a longer and more intensive education [19]. Furthermore, since educational attainment determines the occupational and labour market opportunities individuals encounter during their life course, persisting or increasing trends of education differences in cardiometabolic risk and disease may also be the indirect result of health-adverse working conditions [20] which may be associated with specific cardiometabolic risks and diseases [21].

Thus, on the basis of these previous findings, the main objective of the present study is to investigate the hypothesis stating that the education differences in cardiometabolic risk at the population level in high-income countries have either increased or remained stable for the past 3 decades. More specifically, these education differences will be estimated by investigating the distribution of the following set of cardiometabolic risk factors: (1) BMI, (2) diabetes, (3) systolic and diastolic BP, (4) serum total cholesterol, (5) glycated haemoglobin (HbA1c) and (6) the overall cardiovascular risk score.

The research hypothesis is investigated by analysing data from three large health surveys conducted in the United States (US), England and Scotland, which cover the period between 1992 and 2019.

## Methods

### Data

The present study is based on data from three cross-sectional population health surveys periodically conducted in the United States (US), England and Scotland. In general, the surveys are multi-stage random probability samples of each country and consist of extensive questionnaire and standardised examination data on magnitudes such as BP, anthropometric measurements, blood and urine analytes, medical conditions, prescription medications and socio-economic indicators. The Health Survey for England (HSE) is a stratified random sample of private English households [22]. HSE data are collected directly from persons aged 16 and older in each household. In the present study, HSE data from 1992 to 2019 comprising 28 survey years are considered for the analyses. The National Health and Nutrition Examination Survey (NHANES) is a periodic examination and health survey of the civilian, non-institutionalised population of the United States (US) aged 2 months and older [23]. The present study includes 11 NHANES survey years conducted between 1994 and 2018 in persons aged 16 and older. The third survey is the Scottish Health Survey (SHS), a random-sample of private households in Scotland with a similar scope and structure as the HSE [24]. In the present analyses, SHS data of 14 survey years collected between 1995 and 2019 in individuals 16 years and older are considered.

### Variable harmonisation

In order to ensure the comparability of measurements within and between surveys and to pool the data of the three surveys, the time series of the variables of interest were harmonised by taking into consideration the data collection protocols used in the different surveys over time. The complete list of original variables used for harmonisation in each survey and year is provided in the Additional file 1. *Demographic and anthropometric information*. In the present study demographic data correspond to sex and age of survey participants at the time of interview. Age is categorised within and across surveys as an ordinal variable consisting of 4-year age bands ranging from 16 to 90 years and older (a total of 16 age categories). Anthropometric measures are weight in kilograms and height in metres, which were used to calculate BMI values.

#### Educational attainment

In NHANES, educational attainment is operationalised as the highest grade or level of school respondents’ received, namely, (1) less than high school, (2) high school or some college and (3) college and above.

In the HSE and SHS surveys, the information on education corresponds to the respondent’s highest qualification level and was re-coded as a nominal variable with three categories: university degree or higher, other qualifications, and no qualification. In order to compare educational attainment across surveys, the classification scheme of the International Standard Classification of Education (ISCED) was utilised [25]. The ISCED is a framework for analysing cross-nationally comparable statistics on education and comprises primary, secondary (lower, upper and post-secondary) and tertiary education (bachelor’s, master’s or doctoral level) [26]. Hence, by considering the fact that an incomplete educational level or the lack of qualifications beyond primary education would broadly correspond to a primary or low secondary ISCED educational level, the categories “less than high-school level” in the US or “no qualification level” in England and Scotland were assigned the harmonised category “no qualification”. By the same token, given that college in NHANES and degree level in HSE and SHS correspond to tertiary education in ISCED, both categories were harmonised as “degree level”. Since all other intermediary educational levels in the US, England and Scotland would lie between secondary and up to short-cycle tertiary ISCED educational levels, they were assigned the harmonised category “other qualifications”. Therefore, the comparative analyses reported in the present study are based on a harmonised education variable with three levels (no qualification, degree, and other qualifications), which allows a rather consistent interpretation of results, despite the peculiarities of the single educational titles or certificates of each country.

#### Use of antihypertensive medication

Information on the use of diuretics, beta blockers, calcium-channel blockers and angiotensin-converting-enzyme inhibitors is available in the questionnaire data. In NHANES, except for 1994, prescription medications reported by respondents is coded in a 3-level nested category system (Multum Lexikon) that assigns a therapeutic classification to each reported medication and each ingredient of the medication. In 1994, prescription medications were coded by using a database of the Product Information Branch of the Food and Drug Administration. In the present study, for the 1994 NHANES sample the primary mode of action of up to 14 medications, and for subsequent NHANES survey years, the second level of up to four medications, were used [27]. The information on prescription medications was utilised to identify the aforementioned four major antihypertensive drug classes. In the HSE and SHS surveys, the use of antihypertensive medication is provided as single dichotomous variables corresponding to diuretics, beta blockers, angiotensin-converting-enzyme inhibitors and calcium-channel blockers, which are derived in both surveys from the individual medication codes assigned and collected during standardised interviews (e.g., [28, 29]). The consistency of the self-reported information on antihypertensive drug intake was checked against the information supplied by respondents on whether they were currently taking any medications. Based on the information on the use of antihypertensive medications a dichotomous variable was defined which indicates whether the respondent is under hypertension treatment (yes/no).

#### Blood analytes

Measurements of serum total cholesterol and glycated haemoglobin (HbA1c) were included in the analyses. Total cholesterol was measured by using a cholesterol oxidase enzymatic method as described in detail in the corresponding survey protocols (e.g., [30–32]). HbA1c measurement was performed by boronate affinity high performance liquid chromatography in different analysers over time (e.g., Primus, Tosoh G7 and G8). Until October 2011, HbA1c measurements were calibrated in HSE and SHS by using the Diabetes Control and Complications Trial (DCCT) standards, with successive survey waves using the International Federation of Clinical Chemistry (IFCC) standards [33]. Each survey regularly performs extensive calibration, precision and bias assessments on laboratory results [33, 34].

#### Blood pressure measurements

In each survey, the measurement of systolic and diastolic BP follows a standardised protocol with specific instructions for physicians in NHANES, or study nurses in HSE and SHS (see e.g., [35, 36]). BP measurements are usually taken in the right arm while respondents sit up straight in a comfortable position. Respondents are asked not to eat, smoke, drink alcohol or participate in vigorous activities 30 minutes before BP measurement and to remain quite for about 5 minutes. In the HSE and SHS samples BP measurements are collected by automatic BP devices (e.g., DINAMAP and OMRON HEM PB) and reported as the average of the second and third measurements or average values, respectively [24, 28, 35]. The protocol for BP measurement in NHANES consists of up to four sphygmomanometer readings whose procedure has been described in detail elsewhere [36]. According to the NHANES protocol [36], the BP average of the last two readings is calculated if available. If only one reading was obtained, that reading is used as the average. Readings of diastolic BP less than 30 mmHg were considered missing.

#### Cardiovascular risk scores

The multi-factorial assessment of cardiovascular disease (CVD) risk follows the prediction algorithm for use in primary care proposed by D’Agostino and colleagues, which was obtained with data from the Framingham Heart Study [37]. The CVD scores can be computed by taking into account the regression coefficients $\alpha _j, \, (j = 1, \ldots , p)$ of the proportional-hazards models reported by D’Agostino in which the 10-year risk of a first CVD event is calculated on a set of *p* risk factors. In the present investigation, with the exception of the high-density lipoprotein measurements that were included in the original algorithm of D’Agostino, the CVD risk scores for respondent *i* were based on the respondent’s values for the following risk factors $X_{i,j}$: age (middle value of the age intervals in years), serum total cholesterol mmol/L transformed to mg/dL by the factor 38.7 [38] (chol), systolic BP mmHg (sys), smoking (yes = 1, no = 0) and diabetes (yes, if HbA1c $\ge$ 6.5%; no, otherwise [39]). The 10-year CVD risk score for respondent *i* (Risk$_{CVD,i}$) was calculated by the following equations:

1 $$\begin{aligned} \text{Risk}_{CDV, i} = 1 - S(t)_b ^{ \exp \left( \sum _j ^p \alpha _j X_{i,j} - \sum _j ^p \alpha _j {\bar{X}} \right) } \end{aligned}$$

where

2 $$\begin{aligned} \sum\limits_{j}^{p} {\alpha _{j} } X_{{i,j}} &= 2.32888 \cdot \log ({\text{age}}_{i} ) \\ & \quad + 1.20904 \cdot \log ({\text{chol}}_{i} \cdot 38.7) \\ & \quad + \alpha _{h} \cdot \log ({\text{sys}}_{i} ) + 0.52873 \cdot {\text{smoke}}_{i} \\ & \quad + 0.69154 \cdot {\text{diabetes}}_{i} , \\ \end{aligned}$$

3 $$\begin{aligned} \sum\limits_{j}^{p} {\alpha _{j} } \bar{X} & = 2.32888 \cdot \log ({\text{age}} = 50y) \\ & \quad + 1.20904 \cdot \log ({\text{chol}} = 6.2{\text{mmol/L}} \cdot 38.7) \\ & \quad + 2.76157 \cdot \log ({\text{sys}} = 130{\text{mmHg}}) \\ & \quad + 0.52873 \cdot ({\text{smoke}} = 26\% ) \\ & \quad + 0.69154 \cdot ({\text{diabetes}} = 15\% ), \\ \end{aligned}$$

and

4 $$\begin{aligned} S(t)_b&= {\left\{ \begin{array}{ll} 0.95012, \, \text{women} \\ 0.88936, \, \text{men} \end{array}\right. }\end{aligned}$$

5 $$\begin{aligned} \alpha _h&= {\left\{ \begin{array}{ll} 2.76157, \, \text{if not treated for hypertension} \\ 2.82263, \, \text{otherwise}. \end{array}\right. } \end{aligned}$$

The average quantities ${\bar{X}}$ are comparison values which allow the interpretation of the CVD risk scores for each respondent *i* as the risk difference between one’s own risk and the CVD risk of a person age 50, not treated for hypertension, cholesterol 6.2 mmol/L, systolic BP 130 mmHg and the overall prevalence rates of smoking and diabetes in the pooled sample (26% and 15%, respectively). The function $S(t)_b$ represents the baseline survival probability at $t = 10$ years for females and males, respectively. Even though multiple algorithms have been proposed for calculating the CVD risk, the Framingham risk calculators have shown acceptable agreement performance in risk stratification, even if one or two variables are removed from the calculation formula [40]. Since the regression analysis in the present study is based on the CVD risk scores as a continuous variable, the variability between CVD risk calculators due to the choice of specific cut-off points is avoided and, therefore, the results should be comparable to some extent to the estimates obtained with other CVD risk calculators [40].

#### Sampling design

In order to account for the fact that the sampling design in all surveys is based on some form of cluster and/or stratified sampling, the sampling strata provided in the particular datasets were included to build harmonised sampling strata variables. For the HSE and SHS surveys, the regional units in Scotland and England, and for the NHANES samples, the masked variance pseudo-stratum in each survey year were utilised to take into account the sampling variance of the surveys, respectively.

### Statistical analysis

The research hypothesis is investigated by analysing the temporal trends and education differences of each cardiometabolic risk factor in a series of adjusted linear-mixed models [41]. The random-effects are calculated at the level of the harmonised sampling strata of each survey year. The following adjusted regression models are specified:

6 $$\begin{aligned} Y_{risk} &=\beta _0 + \beta _1 X_{edu} + \beta _2 (X_{edu} \times X_{year}) \\ &\quad + \beta _3 {\mathbf{X}}_{adj} + b_1 \cdot {\mathbf{I}}_{strata} + \epsilon \end{aligned}$$

where $Y_{risk}$ is the dependent variable corresponding to one of the six CVD risk factors, namely, systolic and diastolic BP, BMI, HbA1c (%), cholesterol (mmol/L) and the CVD risk score (%). All dependent variables entered the regression analysis as continuous variables in order to avoid statistical artefacts due to categorisation of genuinely metric magnitudes [42]. With the exception of the CVD risk scores, the dependent variables showed acceptable distribution symmetry around the means. The impact of the skewness in the distribution of the CVD risk scores on the estimates was reduced by performing the regression models on the logarithmic transformation of CVD risk scores. The variable $X_{edu}$ is the educational attainment, $X_{year}$ the survey year and ${\mathbf{X}}_{adj}$ a matrix including the individual characteristics of respondents (sex, age) and, for the analysis of the pooled dataset, dummy variables for each survey, i.e., NHANES (reference category), HSE and SHS. The matrix ${\mathbf{I}}_{strata}$ is an identity matrix used to calculate the random intercepts $b_1$ which vary according to the sampling strata of each survey year. The coefficients $\beta _0, \beta _1, \beta _2, \beta _3$ correspond to the intercept and fixed-effects estimates of the regression models and, finally, $\epsilon$ a vector with residual variation. The coefficients $\beta _1$ express the average differences between the educational attainment categories in the whole observation period. The coefficient $\beta _2$ captures the education vs. time interaction effects, i.e., the relative growth rates of the education categories in comparison to the reference category “other qualifications”. The strata-specific variation takes into account changes in the data collection protocols over time or other unobserved survey-specific characteristics which may have had an impact on the variance of the dependent variables. The reported confidence intervals were estimated at the 99% level to reduce the probability of false positives for small effects in large samples [43]. All statistical analyses were performed with the statistical environment R, especially the estimation routines for linear mixed models implemented in the package lme4.

## Results

### Descriptive statistics

**Fig. 1:**
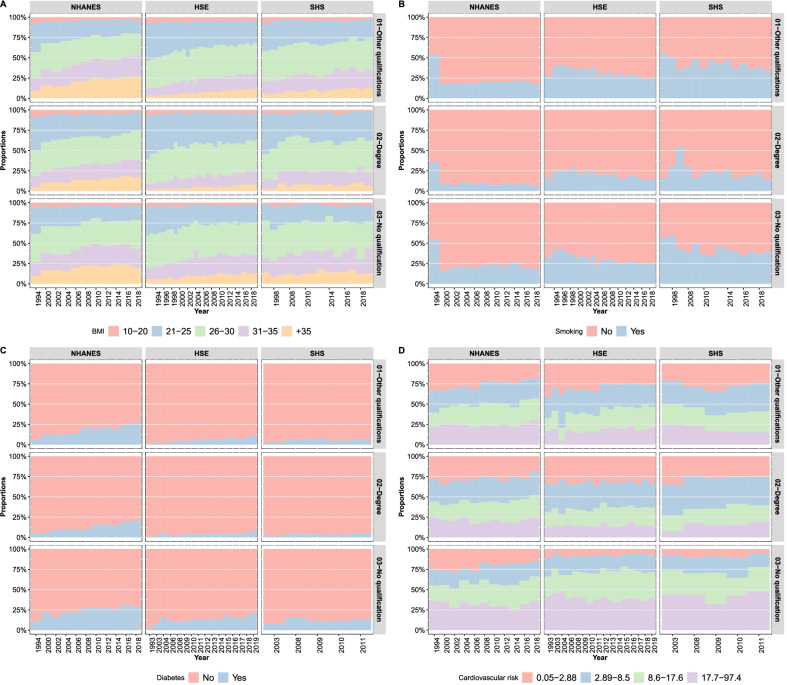
Cardiometabolic risk factors by educational attainment and survey over time. Panel **A**: body-mass-index. Panel **B**: current smoking behaviour. Panel **C**: diabetes prevalence (HbA1c $\ge$ 6.5%). Panel **D**: quartiles of the cardiovascular disease risk score. NHANES: National Health and Nutrition Examination Survey; HSE: Health Survey for England (HSE); SHS: Scottish Health Survey

The pooled dataset comprised a total of 362,275 observations (Table 1). The distribution of the cardiometabolic risk factors across samples reveals some country-specific characteristics. For instance, whereas the percentage of smokers in the HSE and SHS samples is larger than in NHANES, the proportion of overweight and obese individuals in NHANES is substantially larger than in the English and Scottish samples. Average serum total cholesterol, HbA1c and CVD risk scores, on the contrary, seem to be rather similar across samples (Table 1). From the perspective of the time series of the distribution of cardiometabolic risk, Fig. 1 illustrates complex temporal trends of either increasing risks (BMI, diabetes), decreasing (current smoking behaviour) or relatively stable risks (CVD risk scores).

**Table 1:** Descriptive statistics of the surveys for all data collection years combined

Variable	Category	NHANES	HSE	SHS
Sex	Female	53 (78,954)	55 (103,818)	56 (13,593)
	Male	47 (69,586)	45 (85,734)	44 (10,590)
Age	16–29 years	16 (23,886)	18 (33,475)	15 (3556)
	30–49 years	23 (33,620)	35 (65,980)	35 (8458)
	50–64 years	26 (38,089)	23 (44,216)	27 (6608)
	+65 years	36 (52,945)	24 (45,881)	23 (5561)
Body-mass-index (BMI) [kg/m^2^]	10–20	5 (7057)	5 (8931)	4 (924)
	21–25	24 (34,241)	35 (62,358)	31 (6889)
	26–30	32 (45,941)	38 (68,324)	38 (8585)
	31–35	21 (31,163)	16 (27,682)	19 (4159)
	+35	19 (27,105)	6 (11,180)	8 (1849)
Education	Other qualifications	51 (74,278)	55 (104,744)	50 (11,279)
	Degree	19 (27,275)	16 (31,026)	25 (5530)
	No qualification	30 (43,150)	28 (53,782)	25 (5702)
Currently smoking	No	80 (111,578)	69 (84,740)	59 (8545)
	Yes	20 (27,904)	31 (38,929)	41 (6042)
Treated for hypertension	No	57 (85,065)	81 (154,040)	80 (19,301)
	Yes	43 (63,030)	19 (35,422)	20 (4824)
Systolic blood pressure [mmHg]		126.57 (20.41)	131.72 (19.39)	129.96 (19.17)
Diastolic blood pressure [mmHg]		69.62 (12.33)	73.79 (11.92)	73.79 (11.62)
Glycated haemoglobin (HbA1c) [%]		5.92 (1.25)	5.31 (1.05)	5.51 (0.76)
Cholesterol [mmol/L]		4.94 (1.15)	5.52 (1.21)	5.59 (1.17)
CVD risk scores [%]		12.55 (12.78)	11.75 (12.44)	12.75 (12.89)
Sample size		148,540	189,552	24,183

Proportions (%) and frequencies (in parentheses) for categorical variables and means and standard deviations (in parentheses) for continuous variables

**Fig. 2:**
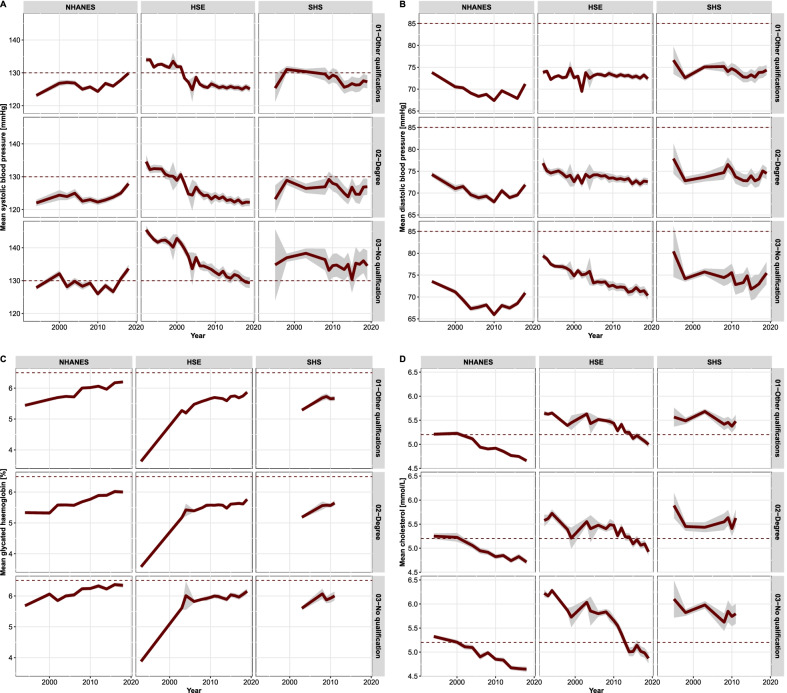
Cardiometabolic risk factors by educational attainment and survey over time. Average values and 99% confidence stripes. Panel **A**: systolic blood pressure (dotted line drawn at the high normal threshold 130 mmHg). Panel **B**: diastolic blood pressure (dotted line drawn at the high normal threshold 85 mmHg) [44]. Panel **C**: average HbA1c (%) (dotted line drawn at the threshold 6.5% [39]). Panel **D**: average serum total cholesterol mmol/L (dotted line drawn at the high-cholesterol threshold 5.2 mmol/L [38])

However, the differences across educational attainment categories in all countries are not only persistent and substantial, but even suggest increasing cardiometabolic health disparities. For instance, diabetes prevalence among individuals without qualification is largest in all samples and, given the population trends toward higher HbA1c measurements, becoming somewhat larger over time, especially in HSE and SHS (Fig. 1). On the other hand, average measurements of systolic and diastolic BP, HbA1c and total cholesterol involve a more complex pattern of temporal and country-specific trends (Fig. 2). For example, average systolic BP has strongly decreased in England, remained relatively stable in Scotland, and increased over time in the US. Nevertheless, despite these country-specific divergent trends, the education differences are quite convergent across samples: Persons with no qualification tend to have higher systolic BP and HbA1c measurements than those with degree or other qualifications (Fig. 2). On the contrary, the average diastolic BP and total cholesterol levels do not seem to be associated with education differences (Fig. 2).

### Regression analysis

The results of the regression analysis provide support to the hypothesis that the education differences in cardiometabolic risk at the population level in high-income countries have either increased or remained stable for the past 3 decades. Support for the stability of education differences is provided by the main effects of the regression coefficients pertaining to educational attainment (Tables 2, 3, 4 and 5). A few instances illustrate this finding: Individuals with degree have lower systolic and diastolic BP and BMI measurements than individuals with other qualifications (e.g., − 2.903 mmHg, 99% CI [− 3.596; − 2.210] systolic BP in NHANES). At the same time, among persons with lower educational attainment the cardiometabolic risks continue to be higher (e.g., 1.870 mmHg , 99% CI [1.342; 2.397] systolic BP in NHANES). To some extent, a similar pattern can be observed for the other cardiometabolic risk factors, namely, HbA1c, total cholesterol and CVD risk scores (Table 3), namely, individuals with degree have consistently lower cardiometabolic risk levels (e.g., − 0.068 99% CI [− 0.103; − 0.033] CVD risk score in HSE), whereas the risk levels among those with no qualification are still larger in comparison to individuals with other qualifications (e.g., 0.285 99% CI  [0.260; 0.309] CVD risk score in HSE). These results point to the stability of the education differences in cardiometabolic risk levels between individuals with degree and those without qualification. The results of the regression models estimated with the pooled dataset confirm these associations found at the country level and indicate a consistent gradient of education differences in the samples (Tables 4 and 5).

**Table 2:** Linear mixed regression models by survey

Variable	SBP	DBP	BMI
*NHANES*			
Intercept	112.891 [112.120; 113.662]	70.438 [69.812; 71.065]	24.136 [23.853; 24.419]
Degree (ref. other qualification)	− 2.903 [− 3.596; − 2.210]	− 0.609 [− 1.042; − 0.175]	− 1.124 [− 1.386; − 0.861]
No qualification	1.870 [1.342; 2.397]	0.213 [− 0.117; 0.544]	0.682 [0.482; 0.882]
Year	− 0.058 [− 0.094; − 0.022]	− 0.387 [− 0.412; − 0.363]	0.197 [0.184; 0.211]
Degree x Year	0.030 [− 0.007; 0.066]	0.054 [0.032; 0.077]	− 0.028 [− 0.042; − 0.015]
No qualification x Year	− 0.029 [− 0.059; 0.001]	− 0.031 [− 0.050; − 0.013]	− 0.041 [− 0.052; − 0.030]
*n*	144703	144703	141791
*m*	148	148	148
*HSE*			
Intercept	127.311 [126.765; 127.857]	66.317 [65.970; 66.664]	23.412 [23.305; 23.520]
Degree (ref. other qualification)	− 1.010 [− 1.432; − 0.589]	1.108 [0.824; 1.393]	− 0.453 [− 0.576; − 0.329]
No qualification	4.181 [3.876; 4.485]	2.984 [2.779; 3.190]	0.500 [0.411; 0.590]
Year	− 0.607 [− 0.621; − 0.592]	− 0.066 [− 0.076; − 0.057]	0.086 [0.082; 0.090]
Degree x Year	0.004 [− 0.022; 0.030]	− 0.059 [− 0.077; − 0.042]	− 0.022 [− 0.029; − 0.014]
No qualification x Year	− 0.223 [− 0.246; − 0.201]	− 0.241 [− 0.257; − 0.226]	0.008 [0.002; 0.015]
*n*	189552	189552	178475
*m*	32	32	32
*SHS*			
Intercept	121.097 [119.719; 122.476]	66.370 [65.458; 67.283]	23.695 [23.314; 24.076]
Degree (ref. other qualification)	− 0.748 [− 2.159; 0.663]	0.355 [− 0.574; 1.284]	0.160 [− 0.280; 0.599]
No qualification	3.118 [1.864; 4.371]	0.389 [− 0.437; 1.214]	0.762 [0.369; 1.154]
Year	− 0.246 [− 0.309; − 0.183]	0.045 [0.003; 0.086]	0.099 [0.082; 0.116]
Degree x Year	0.002 [− 0.072; 0.077]	− 0.027 [− 0.076; 0.022]	− 0.047 [− 0.070; − 0.024]
No qualification x Year	− 0.142 [− 0.218; − 0.066]	− 0.049 [− 0.099; 0.001]	− 0.012 [− 0.035; 0.012]
*n*	22511	22511	20833
*m*	21	21	21

Coefficients $\beta$ and 99% confidence intervals. *m* and *n*: number of random-effect strata levels and sample size, respectively. *SBP, DBP* systolic and diastolic blood pressure. *BMI* body-mass-index

**Table 3:** Linear mixed regressions by survey

Variable	HbA1c	Cholesterol	CVD risk$_{log}$
*NHANES*			
Intercept	4.882 [4.837; 4.927]	4.962 [4.920; 5.005]	− 0.673 [− 0.709; − 0.637]
Degree (ref. other qualification)	− 0.246 [− 0.291; − 0.201]	− 0.069 [− 0.112; − 0.027]	− 0.167 [− 0.196; − 0.138]
No qualification	0.210 [0.176; 0.245]	0.107 [0.075; 0.140]	0.101 [0.079; 0.124]
Year	0.018 [0.015; 0.020]	− 0.021 [− 0.023; − 0.019]	− 0.019 [− 0.021; − 0.017]
Degree x Year	0.003 [0.001; 0.006]	0.003 [0.001; 0.006]	0.002 [0.000; 0.003]
No qualification x Year	− 0.001 [− 0.003; 0.001]	− 0.007 [− 0.009; − 0.006]	− 0.002 [− 0.003; − 0.001]
*n*	138012	136204	127492
*m*	148	148	148
*HSE*			
Intercept	3.824 [3.652; 3.995]	5.073 [5.039; 5.107]	− 0.326 [− 0.351; − 0.301]
Degree (ref. other qualification)	− 0.006 [− 0.045; 0.033]	− 0.011 [− 0.052; 0.029]	− 0.068 [− 0.103; − 0.033]
No qualification	0.054 [0.026; 0.082]	0.281 [0.253; 0.310]	0.285 [0.260; 0.309]
Year	0.047 [0.045; 0.049]	− 0.029 [− 0.031; − 0.028]	− 0.012 [− 0.013; − 0.010]
Degree x Year	− 0.004 [− 0.006; − 0.002]	0.000 [− 0.002; 0.002]	− 0.001 [− 0.003; 0.001]
No qualification x Year	0.005 [0.003; 0.006]	− 0.021 [− 0.023; − 0.019]	− 0.008 [− 0.010; − 0.007]
*n*	66071	88809	39469
*m*	31	32	31
*SHS*			
Intercept	4.257 [4.132; 4.381]	4.666 [4.539; 4.792]	− 0.207 [− 0.343; − 0.072]
Degree (ref. other qualification)	0.210 [0.010; 0.411]	0.018 [− 0.121; 0.158]	0.171 [− 0.054; 0.397]
No qualification	0.244 [0.060; 0.429]	0.110 [− 0.021; 0.241]	0.283 [0.077; 0.489]
Year	0.059 [0.051; 0.066]	0.002 [− 0.005; 0.010]	− 0.005 [− 0.013; 0.003]
Degree x Year	− 0.017 [− 0.029; − 0.004]	− 0.000 [− 0.011; 0.011]	− 0.014 [− 0.028; 0.001]
No qualification x Year	− 0.007 [− 0.020; 0.005]	− 0.006 [− 0.017; 0.005]	− 0.009 [− 0.023; 0.005]
*n*	7168	10781	3723
*m*	21	21	21

Coefficients $\beta$ and 99% confidence intervals. *m* and *n*: number of random-effect strata levels and sample size, respectively. *HbA1c* glycated haemoglobin, *CVD* cardiovascular disease, *Log* logarithm

**Table 4:** Linear mixed regression models with the pooled dataset

Variable	SBP	DBP	BMI
Intercept	121.650 [120.998; 122.302]	63.852 [63.509; 64.194]	25.482 [25.329; 25.635]
Degree (ref. other qualification)	− 1.450 [− 1.804; − 1.096]	0.586 [0.353; 0.818]	− 0.549 [− 0.669; − 0.430]
No qualification	2.788 [2.529; 3.047]	3.050 [2.880; 3.220]	0.591 [0.504; 0.679]
Year	− 0.557 [− 0.571; − 0.544]	− 0.090 [− 0.099; − 0.081]	0.110 [0.106; 0.115]
Degree x Year	− 0.009 [− 0.029; 0.011]	− 0.022 [− 0.036; − 0.009]	− 0.038 [− 0.045; − 0.031]
No qualification x Year	− 0.106 [− 0.122; − 0.089]	− 0.179 [− 0.190; − 0.168]	− 0.017 [− 0.022; − 0.011]
HSE (ref. NHANES)	5.063 [3.649; 6.477]	3.771 [3.068; 4.473]	− 2.245 [− 2.542; − 1.949]
SHS	5.837 [4.134; 7.540]	4.235 [3.385; 5.085]	− 1.869 [− 2.230; − 1.508]
*n*	356766	356766	341099
*m*	201	201	201

Coefficients $\beta$ and 99% confidence intervals. *m* and *n*: number of random-effect strata levels and sample size, respectively. *SBP, DBP* systolic and diastolic blood pressure, *BMI* body-mass-index

**Table 5:** Linear mixed regressions with the pooled dataset

Variable	HbA1c	Cholesterol	CVD risk$_{log}$
Intercept	4.716 [4.663; 4.769]	4.849 [4.822; 4.876]	− 0.658 [− 0.683; − 0.633]
Degree (ref. other qualification)	− 0.171 [− 0.203; − 0.138]	− 0.033 [− 0.062; − 0.005]	− 0.127 [− 0.150; − 0.104]
No qualification	0.160 [0.136; 0.185]	0.268 [0.247; 0.289]	0.136 [0.119; 0.152]
Year	0.033 [0.032; 0.035]	− 0.026 [− 0.027; − 0.025]	− 0.015 [− 0.016; − 0.014]
Degree x Year	0.002 [− 0.000; 0.003]	0.001 [− 0.001; 0.003]	0.000 [− 0.001; 0.001]
No qualification x Year	0.000 [− 0.001; 0.002]	− 0.015 [− 0.017; − 0.014]	− 0.004 [− 0.005; − 0.003]
HSE (ref. NHANES)	− 0.911 [− 1.017; − 0.805]	0.432 [0.392; 0.472]	0.139 [0.103; 0.174]
SHS	v0.277 [− 0.406; − 0.148]	0.537 [0.482; 0.592]	0.252 [0.204; 0.300]
*n*	211251	235794	170684
*m*	200	201	200

Coefficients $\beta$ and 99% confidence intervals. *m* and *n*: number of random-effect strata levels and sample size, respectively. *HbA1c* glycated haemoglobin, *CVD* cardiovascular disease. Log: logarithm

On the other hand, the time and interaction effects estimates reveal that the education differences may increase or decrease, irrespective of whether the general cardiometabolic risk for the whole population increase or decrease. For instance, the regression model for systolic BP with the pooled dataset indicates that the average systolic BP has been decreasing at a rate of − 0.557 mmHg, 99% CI [− 0.571; − 0.544] per year (Table 5). This means that the cardiovascular risk attributable to high systolic BP has actually decreased for the whole population. However, the education differences in systolic BP measurements are still large, with average BP differences between respondents with degree and those with other qualifications amounting to − 1.450 mmHg, 99% CI [− 1.804; − 1.096]. At the same time, the corresponding BP differences between individuals with no qualification in comparison to respondents with other qualifications amount to an increase of about 2.788 mmHg, 99% CI [2.529; 3.047] (Table 5). Nonetheless, taking into account that the interaction effects capture the growth rates of the outcome variable between educational attainment categories, it can be observed that the decrease in systolic BP measurements has been less pronounced among people with other qualifications. This follows from the fact that the interaction effect of education vs. time in the systolic BP model amounted to − 0.009 mmHg, 99% CI [− 0.029; − 0.011] and − 0.106 mmHg, 99% CI [− 0.122; − 0.089] for respondents with degree and respondents with no qualification, respectively. In other words, among persons with degree and no qualification the decrease of systolic BP measurements has been somewhat stronger than among respondents with other qualifications. Similarly, the results of the BMI model with pooled data (Table 5) suggest that the population-based increase in BMI of 0.110 kg/m^2^, 99% CI [0.106; 0.115] per year has been larger among those individuals with other qualifications, for the interaction effects are negative for both university graduates and persons with no qualification, i.e., the trends of weight gain have been relatively steeper among those with other qualifications (− 0.038 kg/m^2^, 99% CI [− 0.045; − 0.031] and − 0.017 kg/m^2^, 99% CI [− 0.022; − 0.011], respectively).

Regarding the demographic characteristics of respondents, the results from all regression models reveal large and consistent age effects across samples (see the Additional file 1 for the complete regression tables). With increasing age the cardiometabolic risk becomes larger for each risk factor considered in the present study. In contrast, the associations of sex and the different risk factors is less consistent across samples. For example, males in the NHANES study have lower BMI levels than females, but in the HSE and SHS studies, on the contrary, they have higher or similar BMI measurements, respectively (Additional file 1). Finally, the analysis with pooled data revealed country-specific patterns of cardiometabolic risk. In the NHANES samples average systolic and diastolic BP measurements, total cholesterol and CVD risk scores are lower than in HSE and SHS. On the contrary, the HbA1c and BMI measurements in the English and Scottish samples are lower than in the US (Table 5).

## Discussion

In the present study the results provided support to the hypothesis that education differences in cardiometabolic risk at the population level in high income countries have either increased or remained stable for the past 3 decades. However, the time series of the cardiometabolic risk factors under consideration show a complex pattern of temporal trends across samples and educational attainment categories. At the population level, average systolic and diastolic BP, serum total cholesterol and the CVD risk scores have decreased over time (Table 5). These trends have been countered by the simultaneous increase of BMI and HbA1c measurements in the last 3 decades which can be observed in each survey and the pooled dataset as well (Tables 3 and 5). At any rate, the education differences have remained large and consistent. In addition, depending on the overall secular trend of specific risk factors in each country, these differences may have widened to some extent. For instance, even though average systolic BP measurements have been decreasing over time, the measurements for individuals in the category “other qualifications” have had a slower decrease rate than individuals with no qualification (− 0.106 mmHg, 99% CI [− 0.122; − 0.089], Table 5). By the same token, the observed increase in BMI measurements has been stronger among those in the “other qualifications” educational category than among individuals with degree or no qualification (− 0.038 kg/m^2^, 99% CI [− 0.045; − 0.031] and − 0.017 kg/m^2^, 99% CI [− 0.022; − 0.011], respectively, Table 5). These findings suggest that education differences in cardiometabolic risk may not only persist, but also widen, even in circumstances of overall improvements of cardiometabolic health, depending on how the cardiometabolic risk levels of individuals in other educational attainment categories change over time.

Moreover, despite the peculiarities in the temporal trends, magnitude and distribution of cardiometabolic risk factors in each country, the pattern of education differences is highly consistent across samples. Even though the surveys considered in the present study are to some extent culturally similar (US, England, Scotland), the persistence of education differences has been also observed in previous research which included more countries in different continents and a large set of chronic diseases such as cancer, mental disorders and several CVD outcomes [21, 45, 46]. Prospective and pooled cross-sectional studies in Finland and South Korea based on population examination data have also documented a temporal trend of accumulating or persisting cardiometabolic risk among individuals with lower education, despite overall improvements of cardiovascular health at the population level over time [12, 47].

From a theoretical perspective, these findings point to the presence of more intricate causal mechanisms arising from the dynamic interaction between the educational level of individuals, their attitudes towards health, their cognitive abilities and, on the other hand, the socioeconomic determinants of health. As discussed by Smith and colleagues (2015), the causal associations between education and health would rather implicate an interaction process whereby individuals adjust their health behaviours given a set of socio-cultural and economic conditions and their own cognitive abilities and attitudes towards health [45]. The result thereof would be reflected in changing patterns of associations between education and health over time and a reshaping of the distribution of the cardiometabolic risk at the population level, with the levels of educational attainment determining the likelihood of adaptive behaviour. For instance, a previous randomised controlled trial assessing the effects of a self-management intervention focusing on self-control of type 2 diabetes or chronic obstructive pulmonary disease revealed that only the more highly educated persons benefited from the intervention [48]. Hence, the education differences would persist even in the presence of population-level improvements if a higher educational attainment tends to facilitate more rapid adaptations in the patterns of health-related factors such as dietary habits, type and frequency of physical activity and attitudes toward health issues.

In a similar vein, even though the present study did not focus on the effects of medical treatment on specific blood analytes (e.g., effects of diabetes medication on HbA1c), it is possible that the education differences estimated in the present study may be related to some extent to the differential utilisation of medical services and treatment options across educational attainment categories or other socioeconomic indicators, as suggested elsewhere [49–51]. Thus, the cardiometabolic risk differences may either increase or decrease depending on the extent to which medical treatment contributes to a relative reduction or relative increase in the cardiometabolic risk profile across educational attainment categories. For instance, among Danish children and adolescents with type 1 diabetes, glycaemic control outcomes were found to be poorer among those individuals whose parents had lower educational attainment [52]. However, research on this topic is limited and requires more efforts to understand how the utilisation of medical services may contribute to the observed education or socioeconomic differences [53].

### Strengths and limitations

The major strength of the present investigation is the use of high-quality population-level data on blood analytes and physical examination measurements which mitigate potential inaccuracies related to self-reported information on health outcomes. In addition, the results are based on large samples covering a time span of about 3 decades and, therefore, facilitate the estimation of time trends of key cardiometabolic risk factors by educational attainment in three high-income countries. To the knowledge of the author, this is the first attempt to analyse simultaneously not only the temporal trends of key factors of cardiometabolic risk, but also the magnitude and temporal trajectories of potential education differences related to that risk in three countries. At the same time, however, there are at least two major limitations which should be taken into account when interpreting the results. First, the analyses did not explicitly consider the potential impact of societal, medical or public health interventions on the distribution of cardiometabolic risk factors in the population. Future research could focus on a detailed assessment of how specific public health and social policies could have exerted some influence on the distribution of single risk factors. Second, the present analyses focused on cardiometabolic risk factors only and, therefore, the clinical significance of the results for the prevalence and incidence of cardiometabolic disease was not addressed. For instance, even though average systolic and diastolic BP and total cholesterol measurements have decreased, BMI and HbA1c have increased across samples, so that the net effect of these diverging trends on cardiometabolic health at the population level (e.g., diabetes incidence, myocardial infarction, stroke, etc.) is to a large extent unknown.

## Conclusions

Education differences in cardiometabolic risk in England, Scotland and the US were found to persist and, in some instances, to increase over time within and across countries. The findings indicate that high educated individuals have had a lower cardiometabolic risk than the lower educated over time in the countries considered. Even in the presence of decreasing systolic and diastolic BP, total cholesterol and CVD risk scores at the population level, the relative education differences could still be observed rather consistently in all samples. At the same time, the increase in cardiometabolic risk due to higher BMI and HbA1c at the population level has affected more frequently individuals with lower education. The results suggest a more complex pattern of associations between education and health which may be due to education-dependent processes related to behavioural, cognitive and attitudinal modification and adaptation to changing socio-cultural conditions.

## Supplementary Information

**Additional file 1.** List of original survey variables and complete regression tables.

## Data Availability

The data of HSE and SHS are publicly available on request from the UK Data Service established by the Economic and Social Research Council in the United Kingdom https://ukdataservice.ac.uk/. NHANES data are publicly available from the Centers for Disease Prevention and Control https://www.cdc.gov/nchs/nhanes/.

## Abbreviations

BP: Blood pressure
HbA1c: Glycated haemoglobin 1c
BMI: Body-mass-index
CVD: Cardiovascular disease
HSE: The Health Survey for England
NHANES: The National Health and Nutrition Examination Survey
SHS: Scottish Health Survey.
