# Supplementary material for: Education differences in cardiometabolic risk in England, Scotland and the United States between 1992 and 2019
Source: BMC Cardiovasc Disord. 2022 Jun 2;22:247. doi: 10.1186/s12872-022-02681-y (PMC9161563; doi:10.1186/s12872-022-02681-y)
Supplement: Supplementary file 1 — Additional file 1. List of original survey variables and complete regression tables. [file 12872_2022_2681_MOESM1_ESM.pdf]

Education differences in cardiometabolic risk in England, Scotland and the United States between 1992 and 2019

Supplementary file

1 Variable harmonisation

Table 1: Harmonised and original variables by survey and year. Antihypertensive medications in NHANES in 1994 includes 14 variables starting with `hax9dg`. In HSE 1992, antihypertensive medications were identified from 10 variables coding for up to 10 medications. Calculation of diastolic and systolic blood pressure in NHANES 2000-2018 was based on the single measurements recorded in the variables starting with `bpxdi` and `bpxsy`. Blood pressure measurements in SHS between 2003-2019 were calculated with the variables starting with `dias` and `sys`.

Year	Harmonised variable	NHANES	HSE	SHS
1992	age		age	
1992	antihypertensives		medications	
1992	antihypertensives			
1992	antihypertensives			
1992	antihypertensives			
1992	cholesterol		cholest	
1992	diastolic		diastolic	
1992	height		height	
1992	sex		sex	
1992	smoking		ciggrp	
1992	strata		rhaarea	
1992	systolic		systolic	
1992	weight		weight	
1993	age		age	
1993	antihypertensives		diur	
1993	antihypertensives		beta	
1993	antihypertensives		ace	
1993	antihypertensives		calc	
1993	cholesterol		cholest	
1993	diastolic		newdiast	
1993	glycated haemoglobin		glyco	
1993	height		height	
1993	sex		sex	
1993	smoking		ciggrp	
1993	strata		rha14	

Continues on the next page ... . . .

Year	Harmonised variable	NHANES	HSE	SHS
1993	systolic		newsyst	
1993	weight		weight	
1994	age	hsageir	age	
1994	antihypertensives	hax9dg	cvdmed01	
1994	antihypertensives		cvdmed02	
1994	antihypertensives		cvdmed04	
1994	antihypertensives		cvdmed07	
1994	cholesterol	tcpsi		
1994	cholesterol		cholest	
1994	diastolic	pepmnk5r	newdiast	
1994	glycated haemoglobin	ghp		
1994	height	bmpht	height	
1994	sex	hssex	sex	
1994	smoking	har3	smokenow	
1994	strata	sdpstra6	gor	
1994	systolic	pepmnk1r	newsyst	
1994	weight	bmpwt	weight	
1995	age		age	respage
1995	antihypertensives		diur	cvdmed01
1995	antihypertensives		beta	beta
1995	antihypertensives		aceinh	aceinh
1995	antihypertensives		calciumb	calciumb
1995	cholesterol			cholv
1995	diastolic		newdiast	diastol
1995	height		height	height
1995	sex		sex	respsex
1995	smoking		smokenow	smokenow
1995	strata		gor	strata
1995	systolic		newsyst	systol
1995	weight		weight	weight
1996	age		age	
1996	antihypertensives		diur	
1996	antihypertensives		beta	
1996	antihypertensives		aceinh	
1996	antihypertensives		calciumb	
1996	diastolic		newdiast	
1996	height		height	
1996	sex		sex	
1996	smoking		smokenow	
1996	strata		gor	
1996	systolic		newsyst	
1996	weight		weight	
1997	age		age	
1997	antihypertensives		diur	
1997	antihypertensives		beta	
1997	antihypertensives		aceinh	
1997	antihypertensives		calciumb	
1997	diastolic		newdiast	
1997	height		height	

Continues on the next page ... . . .

Year	Harmonised variable	NHANES	HSE	SHS
1997	sex		sex	
1997	smoking		cignow	
1997	strata		gor	
1997	systolic		sysval	
1997	weight		weight	
1998	age		age	age
1998	antihypertensives		diur	diur
1998	antihypertensives		beta	beta
1998	antihypertensives		aceinh	aceinh
1998	antihypertensives		calciumb	calciumb
1998	cholesterol		cholval	
1998	cholesterol			cholval
1998	diastolic		newdiast	diaval
1998	height		height	height
1998	sex		sex	sex
1998	smoking		cignow	smokenow
1998	strata		gor	region
1998	systolic		sysval	sysval
1998	weight		weight	weight
1999	age		age	
1999	antihypertensives		diur	
1999	antihypertensives		beta	
1999	antihypertensives		aceinh	
1999	antihypertensives		calciumb	
1999	cholesterol		cholval	
1999	diastolic		newdiast	
1999	height		height	
1999	sex		sex	
1999	smoking		cignow	
1999	strata		gor	
1999	systolic		sysval	
1999	weight		weight	
2000	age	ridageyr	age	
2000	antihypertensives	rxddci1b	diur	
2000	antihypertensives	rxddci1b	beta	
2000	antihypertensives	rxddci1b	aceinh	
2000	antihypertensives	rxddci1b	calciumb	
2000	cholesterol	lbdtcsi		
2000	diastolic	bpxdi	newdiast	
2000	glycated haemoglobin	lbxgh		
2000	height	bmxht	height	
2000	sex	riagendr	sex	
2000	smoking	smq040	cignow	
2000	strata	sdmvstra	gor	
2000	systolic	bpxsy	sysval	
2000	weight	bmxwt	weight	
2001	age		age	
2001	antihypertensives		diur	
2001	antihypertensives		beta	

Continues on the next page ... . . .

Year	Harmonised variable	NHANES	HSE	SHS
2001	antihypertensives		aceinh	
2001	antihypertensives		calciumb	
2001	diastolic		newdiast	
2001	height		height	
2001	sex		sex	
2001	smoking		cignow	
2001	strata		gora	
2001	systolic		sysval	
2001	weight		weight	
2002	age	ridageyr	age	
2002	antihypertensives	rxddci1b	diur	
2002	antihypertensives	rxddci1b	beta	
2002	antihypertensives	rxddci1b	aceinh	
2002	antihypertensives	rxddci1b	calciumb	
2002	cholesterol	lbdtcsi		
2002	diastolic	bpxdi	newdiast	
2002	glycated haemoglobin	lbxgh		
2002	height	bmxht	height	
2002	sex	riagendr	sex	
2002	smoking	smq040	cignow	
2002	strata	sdmvstra	gor	
2002	systolic	bpxsy	sysval	
2002	weight	bmxwt	weight	
2003	age		age	age
2003	antihypertensives		diur	diur
2003	antihypertensives		beta	beta
2003	antihypertensives		aceinh	aceinh
2003	antihypertensives		calciumb	calciumb
2003	cholesterol		cholval	
2003	cholesterol			cholval
2003	diastolic		omdiaval	dias
2003	glycated haemoglobin		glyhbval	
2003	glycated haemoglobin			glyhb
2003	height		height	height
2003	sex		sex	sex
2003	smoking		cignow	smokenow
2003	strata		gor	strata
2003	systolic		omsysval	sys
2003	weight		weight	weight
2004	age	ridageyr	age	
2004	antihypertensives	rxddci1b	diur	
2004	antihypertensives	rxddci1b	beta	
2004	antihypertensives	rxddci1b	aceinh	
2004	antihypertensives	rxddci1b	calciumb	
2004	cholesterol	lbdtcsi		
2004	cholesterol		cholval	
2004	diastolic	bpxdi	omdiaval	
2004	glycated haemoglobin	lbxgh		
2004	glycated haemoglobin		glyhbval	

Continues on the next page ... . . .

Year	Harmonised variable	NHANES	HSE	SHS
2004	height	bmxht	height	
2004	sex	riagendr	sex	
2004	smoking	smq040	cignow	
2004	strata	sdmvstra	gor	
2004	systolic	bpxsy	omsysval	
2004	weight	bmxwt	weight	
2005	age		age	
2005	antihypertensives		diur	
2005	antihypertensives		beta	
2005	antihypertensives		aceinh	
2005	antihypertensives		calciumb	
2005	diastolic		omdiaval	
2005	height		height	
2005	sex		sex	
2005	smoking		cignow	
2005	strata		gor	
2005	systolic		omsysval	
2005	weight		weight	
2006	age	ridageyr	age	
2006	antihypertensives	rxddci1b	diur	
2006	antihypertensives	rxddci1b	beta	
2006	antihypertensives	rxddci1b	aceinh	
2006	antihypertensives	rxddci1b	calciumb	
2006	cholesterol	lbdtcsi		
2006	cholesterol		cholval	
2006	diastolic	bpxdi	omdiaval	
2006	glycated haemoglobin	lbxgh		
2006	glycated haemoglobin		glyhbval	
2006	height	bmxht	height	
2006	sex	riagendr	sex	
2006	smoking	smq040	cignow	
2006	strata	sdmvstra	gor06	
2006	systolic	bpxsy	omsysval	
2006	weight	bmxwt	weight	
2007	age		age	
2007	antihypertensives		diur	
2007	antihypertensives		beta	
2007	antihypertensives		aceinh	
2007	antihypertensives		calciumb	
2007	diastolic		omdiaval	
2007	height		height	
2007	sex		sex	
2007	smoking		cignow	
2007	strata		gor7	
2007	systolic		omsysval	
2007	weight		weight	
2008	age	ridageyr	age	age
2008	antihypertensives	rxddci1b	diur	diur
2008	antihypertensives	rxddci1b	beta	beta

Continues on the next page ... . . .

Year	Harmonised variable	NHANES	HSE	SHS
2008	antihypertensives	rxddci1b	aceinh	aceinh
2008	antihypertensives	rxddci1b	calciumb	calciumb
2008	cholesterol	lbdtcsi		
2008	cholesterol		cholval	
2008	cholesterol			cholval
2008	diastolic	bpxdi	omdiaval	dias
2008	glycated haemoglobin	lbxgh		
2008	glycated haemoglobin		glyhbval	
2008	glycated haemoglobin			glyhb
2008	height	bmxht	height	height
2008	sex	riagendr	sex	sex
2008	smoking	smq040	cignow	smokenow
2008	strata	sdmvstra	gor	strata
2008	systolic	bpxsy	omsysval	sys
2008	weight	bmxwt	weight	weight
2009	age		age	age
2009	antihypertensives		diur	diur
2009	antihypertensives		beta	beta
2009	antihypertensives		aceinh	aceinh
2009	antihypertensives		calciumb	calciumb
2009	cholesterol		cholval	
2009	cholesterol			cholval
2009	diastolic		omdiaval	dias
2009	glycated haemoglobin		glyhbval	
2009	glycated haemoglobin			glyhb
2009	height		height	height
2009	sex		sex	sex
2009	smoking		cignow	smokenow
2009	strata		gor07	strata
2009	systolic		omsysval	sys
2009	weight		weight	weight
2010	age	ridageyr	age	age
2010	antihypertensives	rxddci1b	diur	diur
2010	antihypertensives	rxddci1b	beta	beta
2010	antihypertensives	rxddci1b	aceinh	aceinh
2010	antihypertensives	rxddci1b	calciumb	calciumb
2010	cholesterol	lbdtcsi		
2010	cholesterol		cholval	
2010	cholesterol			cholval
2010	diastolic	bpxdi	omdiaval	dias
2010	glycated haemoglobin	lbxgh		
2010	glycated haemoglobin		glyhbval	
2010	glycated haemoglobin			glyhb
2010	height	bmxht	height	height
2010	sex	riagendr	sex	sex
2010	smoking	smq040	cignow	smokenow
2010	strata	sdmvstra	gor1	strata
2010	systolic	bpxsy	omsysval	sys
2010	weight	bmxwt	weight	weight

Continues on the next page ... . . .

Year	Harmonised variable	NHANES	HSE	SHS
2011	age		age	age
2011	antihypertensives		diur	diur
2011	antihypertensives		beta	beta
2011	antihypertensives		aceinh	aceinh
2011	antihypertensives		calciumb	calciumb
2011	cholesterol		cholvalold	
2011	cholesterol			cholval
2011	diastolic		omdiaval	dias
2011	glycated haemoglobin		glyhbval	
2011	glycated haemoglobin			glyhb
2011	height		height	height
2011	sex		sex	sex
2011	smoking		cignow	smokenow
2011	strata		gor1	strata
2011	systolic		omsysval	sys
2011	weight		weight	weight
2012	age	ridageyr	age	
2012	antihypertensives	rxddci1b	diur2	
2012	antihypertensives	rxddci1b	beta2	
2012	antihypertensives	rxddci1b	aceinh2	
2012	antihypertensives	rxddci1b	calciumb2	
2012	cholesterol	lbdtcsi		
2012	cholesterol		cholval12	
2012	diastolic	bpxdi	omdiaval	
2012	glycated haemoglobin	lbxgh		
2012	glycated haemoglobin		glyhbval	
2012	height	bmxht	height	
2012	sex	riagendr	sex	
2012	smoking	smq040	cignow	
2012	strata	sdmvstra	gor1	
2012	systolic	bpxsy	omsysval	
2012	weight	bmxwt	weight	
2013	age		age	age
2013	antihypertensives		diur2	diur
2013	antihypertensives		beta2	beta
2013	antihypertensives		aceinh2	aceinh
2013	antihypertensives		calciumb2	calciumb
2013	cholesterol		cholval12	
2013	diastolic		omdiaval	dias
2013	glycated haemoglobin		glyhbval	
2013	height		height	height
2013	sex		sex	sex
2013	smoking		cignow	smokenow
2013	strata		gor1	strata
2013	systolic		omsysval	sys
2013	weight		weight	weight
2014	age	ridageyr	age	age
2014	antihypertensives	rxddci1b	diur2	diur
2014	antihypertensives	rxddci1b	beta2	beta

Continues on the next page ... . . .

Year	Harmonised variable	NHANES	HSE	SHS
2014	antihypertensives	rxddci1b	aceinh2	aceinh
2014	antihypertensives	rxddci1b	calciumb2	calciumb
2014	cholesterol	lbdtcsi		
2014	cholesterol		cholval12	
2014	diastolic	bpxdi	omdiaval	dias
2014	glycated haemoglobin	lbxgh		
2014	glycated haemoglobin		glyhbval	
2014	height	bmxht	height	height
2014	sex	riagendr	sex	sex
2014	smoking	smq040	cignow	smokenow
2014	strata	sdmvstra	gor1	strata
2014	systolic	bpxsy	omsysval	sys
2014	weight	bmxwt	weight	weight
2015	age		age	age
2015	antihypertensives		diur2	diur
2015	antihypertensives		beta2	beta
2015	antihypertensives		aceinh2	aceinh
2015	antihypertensives		calciumb2	calciumb
2015	cholesterol		cholval1a	
2015	diastolic		omdiaval	dias
2015	glycated haemoglobin		glyhbvala	
2015	height		height	height
2015	sex		sex	sex
2015	smoking		cignow	smokenow
2015	strata		gor1	strata
2015	systolic		omsysval	sys
2015	weight		weight	weight
2016	age	ridageyr	age	age
2016	antihypertensives	rxddci1b	diur2	diur
2016	antihypertensives	rxddci1b	beta2	beta
2016	antihypertensives	rxddci1b	aceinh2	aceinh
2016	antihypertensives	rxddci1b	calciumb2	calciumb
2016	cholesterol	lbdtcsi		
2016	cholesterol		cholval1a	
2016	diastolic	bpxdi	omdiaval	dias
2016	glycated haemoglobin	lbxgh		
2016	glycated haemoglobin		glyhbvala	
2016	height	bmxht	height	height
2016	sex	riagendr	sex	sex
2016	smoking	smq040	cignow	smokenow
2016	strata	sdmvstra	gor1	strata
2016	systolic	bpxsy	omsysval	sys
2016	weight	bmxwt	weight	weight
2017	age		age	age
2017	antihypertensives		diur2	diur
2017	antihypertensives		beta2	beta
2017	antihypertensives		aceinh2	aceinh
2017	antihypertensives		calciumb2	calciumb
2017	cholesterol		cholval1a	

Continues on the next page ... ...

Year	Harmonised variable	NHANES	HSE	SHS
2017	diastolic		omdiaval	dias
2017	glycated haemoglobin		glyhbvala	
2017	height		height	height
2017	sex		sex	sex
2017	smoking		cignow	smokenow
2017	strata		gor1	strata
2017	systolic		omsysval	sys
2017	weight		weight	weight
2018	age	ridageyr	age	age
2018	antihypertensives	rxddci1b	diur2	diur
2018	antihypertensives	rxddci1b	beta2	beta
2018	antihypertensives	rxddci1b	aceinh2	aceinh
2018	antihypertensives	rxddci1b	calciumb2	calciumb
2018	cholesterol	lbdtcsi		
2018	cholesterol		cholval1a	
2018	diastolic	bpxdi	omdiaval	dias
2018	glycated haemoglobin	lbxgh		
2018	glycated haemoglobin		glyhbvala	
2018	height	bmxht	height	height
2018	sex	riagendr	sex	sex
2018	smoking	smq040	cignow	smokenow
2018	strata	sdmvstra	gor1	strata
2018	systolic	bpxsy	omsysval	sys
2018	weight	bmxwt	weight	weight
2019	age		age	age
2019	antihypertensives		diur2	diur
2019	antihypertensives		beta2	beta
2019	antihypertensives		aceinh2	aceinh
2019	antihypertensives		calciumb2	calciumb
2019	cholesterol		cholval1a	
2019	diastolic		omdiaval	dias
2019	glycated haemoglobin		glyhbvala	
2019	height		height	height
2019	sex		sex	sex
2019	smoking		cignow	smokenow
2019	strata		gor1	strata
2019	systolic		omsysval	sys
2019	weight		weight	weight

2 Regression tables

Table 2a: Linear mixed regression models by survey. Coefficients β and 99% confidence intervals. m and n: number of random-effect strata levels and sample size, respectively. σ: standard deviation of the random intercepts and residuals. SBP, DBP: systolic and diastolic blood pressure. BMI: body-mass-index

Variable	SBP	DBP	BMI
**NAHNES**			
Intercept	112.891 [112.120; 113.662]	70.438 [69.812; 71.065]	24.136 [23.853; 24.419]
Degree (ref. other qualification)	-2.903 [-3.596; -2.210]	-0.609 [-1.042; -0.175]	-1.124 [-1.386; -0.861]
No qualification	1.870 [1.342; 2.397]	0.213 [-0.117; 0.544]	0.682 [0.482; 0.882]
Year	-0.058 [-0.094; -0.022]	-0.387 [-0.412; -0.363]	0.197 [0.184; 0.211]
Degree x Year	0.030 [-0.007; 0.066]	0.054 [0.032; 0.077]	-0.028 [-0.042; -0.015]
No qualification x Year	-0.029 [-0.059; 0.001]	-0.031 [-0.050; -0.013]	-0.041 [-0.052; -0.030]
Male (ref. Female)	0.137 [-0.051; 0.326]	2.176 [2.059; 2.294]	-0.978 [-1.050; -0.906]
30-49 years (ref. 16-29 years)	7.283 [6.974; 7.592]	9.264 [9.071; 9.457]	3.650 [3.533; 3.767]
50-64 years	16.684 [16.380; 16.989]	8.485 [8.295; 8.675]	4.254 [4.139; 4.370]
65 years and older	24.241 [23.951; 24.530]	1.282 [1.102; 1.463]	2.278 [2.168; 2.388]
n	144703	144703	141791
m	148	148	148
σ_{strata}	2.45	2.7	0.86
$\sigma_{residual}$	18.19	11.36	6.85
**HSE**			
Intercept	127.311 [126.765; 127.857]	66.317 [65.970; 66.664]	23.412 [23.305; 23.520]
Degree (ref. other qualification)	-1.010 [-1.432; -0.589]	1.108 [0.824; 1.393]	-0.453 [-0.576; -0.329]
No qualification	4.181 [3.876; 4.485]	2.984 [2.779; 3.190]	0.500 [0.411; 0.590]
Year	-0.607 [-0.621; -0.592]	-0.066 [-0.076; -0.057]	0.086 [0.082; 0.090]
Degree x Year	0.004 [-0.022; 0.030]	-0.059 [-0.077; -0.042]	-0.022 [-0.029; -0.014]
No qualification x Year	-0.223 [-0.246; -0.201]	-0.241 [-0.257; -0.226]	0.008 [0.002; 0.015]
Male (ref. Female)	5.060 [4.909; 5.210]	2.651 [2.549; 2.752]	0.372 [0.328; 0.417]
30-49 years (ref. 16-29 years)	3.025 [2.806; 3.245]	7.336 [7.188; 7.484]	2.186 [2.122; 2.251]
50-64 years	13.124 [12.882; 13.366]	11.295 [11.132; 11.459]	3.058 [2.987; 3.129]
65 years and older	21.836 [21.584; 22.088]	8.521 [8.350; 8.691]	2.519 [2.445; 2.594]
n	189552	189552	178475
m	32	32	32
σ_{strata}	1.36	0.85	0.21
$\sigma_{residual}$	16.53	11.15	4.73
**SHS**			
Intercept	121.097 [119.719; 122.476]	66.370 [65.458; 67.283]	23.695 [23.314; 24.076]
Degree (ref. other qualification)	-0.748 [-2.159; 0.663]	0.355 [-0.574; 1.284]	0.160 [-0.280; 0.599]
No qualification	3.118 [1.864; 4.371]	0.389 [-0.437; 1.214]	0.762 [0.369; 1.154]
Year	-0.246 [-0.309; -0.183]	0.045 [0.003; 0.086]	0.099 [0.082; 0.116]
Degree x Year	0.002 [-0.072; 0.077]	-0.027 [-0.076; 0.022]	-0.047 [-0.070; -0.024]
No qualification x Year	-0.142 [-0.218; -0.066]	-0.049 [-0.099; 0.001]	-0.012 [-0.035; 0.012]
Male (ref. Female)	4.842 [4.396; 5.288]	1.695 [1.402; 1.989]	0.036 [-0.103; 0.175]
30-49 years (ref. 16-29 years)	3.592 [2.871; 4.313]	6.281 [5.806; 6.755]	2.224 [2.000; 2.448]
50-64 years	14.650 [13.900; 15.400]	9.935 [9.442; 10.429]	3.068 [2.835; 3.301]
65 years and older	23.235 [22.447; 24.023]	5.889 [5.370; 6.407]	2.656 [2.410; 2.901]
n	22511	22511	20833
m	21	21	21
σ_{strata}	1.07	0.72	0.22
$\sigma_{residual}$	16.9	11.13	5.08

Table 2b: Linear mixed regressions by survey. Coefficients β and 99% confidence intervals. m and n: number of random-effect strata levels and sample size, respectively. σ: standard deviation of the random intercepts and residuals. HbA1c: glycated haemoglobin. CVD: cardiovascular disease. Log: logarithm.

**Variable**	**HbA1c**	**Cholesterol**	**CVD risk$_{log}$**
**NAHNES**			
Intercept	4.882 [4.837; 4.927]	4.962 [4.920; 5.005]	-0.673 [-0.709; -0.637]
Degree (ref. other qualification)	-0.246 [-0.291; -0.201]	-0.069 [-0.112; -0.027]	-0.167 [-0.196; -0.138]
No qualification	0.210 [0.176; 0.245]	0.107 [0.075; 0.140]	0.101 [0.079; 0.124]
Year	0.018 [0.015; 0.020]	-0.021 [-0.023; -0.019]	-0.019 [-0.021; -0.017]
Degree x Year	0.003 [0.001; 0.006]	0.003 [0.001; 0.006]	0.002 [0.000; 0.003]
No qualification x Year	-0.001 [-0.003; 0.001]	-0.007 [-0.009; -0.006]	-0.002 [-0.003; -0.001]
Male (ref. Female)	0.093 [0.081; 0.106]	-0.332 [-0.344; -0.321]	0.742 [0.735; 0.749]
30-49 years (ref. 16-29 years)	0.463 [0.443; 0.483]	0.661 [0.642; 0.680]	1.905 [1.894; 1.917]
50-64 years	0.990 [0.970; 1.010]	0.745 [0.726; 0.764]	2.990 [2.978; 3.001]
65 years and older	0.906 [0.887; 0.925]	0.437 [0.419; 0.455]	3.522 [3.511; 3.533]
n	138012	136204	127492
m	148	148	148
σ_{strata}	0.12	0.11	0.11
$\sigma_{residual}$	1.17	1.09	0.63
**HSE**			
Intercept	3.824 [3.652; 3.995]	5.073 [5.039; 5.107]	-0.326 [-0.351; -0.301]
Degree (ref. other qualification)	-0.006 [-0.045; 0.033]	-0.011 [-0.052; 0.029]	-0.068 [-0.103; -0.033]
No qualification	0.054 [0.026; 0.082]	0.281 [0.253; 0.310]	0.285 [0.260; 0.309]
Year	0.047 [0.045; 0.049]	-0.029 [-0.031; -0.028]	-0.012 [-0.013; -0.010]
Degree x Year	-0.004 [-0.006; -0.002]	0.000 [-0.002; 0.002]	-0.001 [-0.003; 0.001]
No qualification x Year	0.005 [0.003; 0.006]	-0.021 [-0.023; -0.019]	-0.008 [-0.010; -0.007]
Male (ref. Female)	0.082 [0.071; 0.093]	-0.120 [-0.135; -0.106]	0.906 [0.894; 0.917]
30-49 years (ref. 16-29 years)	0.183 [0.166; 0.201]	0.713 [0.691; 0.735]	1.398 [1.380; 1.416]
50-64 years	0.469 [0.451; 0.488]	1.277 [1.253; 1.301]	2.457 [2.438; 2.477]
65 years and older	0.592 [0.573; 0.612]	1.138 [1.113; 1.164]	3.005 [2.985; 3.026]
n	66071	88809	39469
m	31	32	31
σ_{strata}	0.48	0.06	0.03
$\sigma_{residual}$	0.71	1.09	0.58
**SHS**			
Intercept	4.257 [4.132; 4.381]	4.666 [4.539; 4.792]	-0.207 [-0.343; -0.072]
Degree (ref. other qualification)	0.210 [0.010; 0.411]	0.018 [-0.121; 0.158]	0.171 [-0.054; 0.397]
No qualification	0.244 [0.060; 0.429]	0.110 [-0.021; 0.241]	0.283 [0.077; 0.489]
Year	0.059 [0.051; 0.066]	0.002 [-0.005; 0.010]	-0.005 [-0.013; 0.003]
Degree x Year	-0.017 [-0.029; -0.004]	-0.000 [-0.011; 0.011]	-0.014 [-0.028; 0.001]
No qualification x Year	-0.007 [-0.020; 0.005]	-0.006 [-0.017; 0.005]	-0.009 [-0.023; 0.005]
Male (ref. Female)	0.029 [-0.003; 0.062]	-0.117 [-0.158; -0.076]	0.895 [0.859; 0.932]
30-49 years (ref. 16-29 years)	0.180 [0.124; 0.236]	0.753 [0.686; 0.820]	1.330 [1.266; 1.394]
50-64 years	0.455 [0.396; 0.513]	1.375 [1.304; 1.446]	2.314 [2.248; 2.381]
65 years and older	0.641 [0.580; 0.703]	1.269 [1.191; 1.348]	2.903 [2.831; 2.974]
n	7168	10781	3723
m	21	21	21
σ_{strata}	0.02	0.07	0.02
$\sigma_{residual}$	0.69	1.08	0.56

Table 3a: Linear mixed regression models with the pooled dataset. Coefficients β and 99% confidence intervals. m and n: number of random-effect strata levels and sample size, respectively. σ: standard deviation of the random intercepts and residuals. SBP, DBP: systolic and diastolic blood pressure. BMI: body-mass-index

Variable	SBP	DBP	BMI
Intercept	121.650 [120.998; 122.302]	63.852 [63.509; 64.194]	25.482 [25.329; 25.635]
Degree (ref. other qualification)	-1.450 [-1.804; -1.096]	0.586 [0.353; 0.818]	-0.549 [-0.669; -0.430]
No qualification	2.788 [2.529; 3.047]	3.050 [2.880; 3.220]	0.591 [0.504; 0.679]
Year	-0.557 [-0.571; -0.544]	-0.090 [-0.099; -0.081]	0.110 [0.106; 0.115]
Degree x Year	-0.009 [-0.029; 0.011]	-0.022 [-0.036; -0.009]	-0.038 [-0.045; -0.031]
No qualification x Year	-0.106 [-0.122; -0.089]	-0.179 [-0.190; -0.168]	-0.017 [-0.022; -0.011]
Male (ref. Female)	3.031 [2.917; 3.146]	2.357 [2.282; 2.432]	-0.222 [-0.261; -0.183]
30-49 years (ref. 16-29 years)	4.612 [4.437; 4.788]	7.703 [7.588; 7.818]	2.713 [2.654; 2.772]
50-64 years	14.721 [14.536; 14.905]	10.018 [9.897; 10.139]	3.573 [3.511; 3.635]
65 years and older	22.822 [22.639; 23.005]	4.815 [4.695; 4.935]	2.345 [2.283; 2.407]
HSE (ref. NHANES)	5.063 [3.649; 6.477]	3.771 [3.068; 4.473]	-2.245 [-2.542; -1.949]
SHS	5.837 [4.134; 7.540]	4.235 [3.385; 5.085]	-1.869 [-2.230; -1.508]
n	356766	356766	341099
m	201	201	201
σ_{strata}	3.67	1.81	0.76
$\sigma_{residual}$	17.34	11.38	5.75

Table 3b: Linear mixed regressions with the pooled dataset. Coefficients β and 99% confidence intervals. m and n: number of random-effect strata levels and sample size, respectively. σ: standard deviation of the random intercepts and residuals. HbA1c: glycated haemoglobin. CVD: cardiovascular disease. Log: logarithm.

Variable	HbA1c	Cholesterol	CVD risk$_{log}$
Intercept	4.716 [4.663; 4.769]	4.849 [4.822; 4.876]	-0.658 [-0.683; -0.633]
Degree (ref. other qualification)	-0.171 [-0.203; -0.138]	-0.033 [-0.062; -0.005]	-0.127 [-0.150; -0.104]
No qualification	0.160 [0.136; 0.185]	0.268 [0.247; 0.289]	0.136 [0.119; 0.152]
Year	0.033 [0.032; 0.035]	-0.026 [-0.027; -0.025]	-0.015 [-0.016; -0.014]
Degree x Year	0.002 [-0.000; 0.003]	0.001 [-0.001; 0.003]	0.000 [-0.001; 0.001]
No qualification x Year	0.000 [-0.001; 0.002]	-0.015 [-0.017; -0.014]	-0.004 [-0.005; -0.003]
Male (ref. Female)	0.090 [0.081; 0.098]	-0.248 [-0.257; -0.239]	0.783 [0.777; 0.789]
30-49 years (ref. 16-29 years)	0.381 [0.366; 0.395]	0.644 [0.630; 0.659]	1.778 [1.768; 1.788]
50-64 years	0.808 [0.793; 0.823]	0.967 [0.953; 0.982]	2.863 [2.853; 2.873]
65 years and older	0.799 [0.785; 0.813]	0.685 [0.670; 0.699]	3.406 [3.397; 3.416]
HSE (ref. NHANES)	-0.911 [-1.017; -0.805]	0.432 [0.392; 0.472]	0.139 [0.103; 0.174]
SHS	-0.277 [-0.406; -0.148]	0.537 [0.482; 0.592]	0.252 [0.204; 0.300]
n	211251	235794	170684
m	200	201	200
σ_{strata}	0.27	0.1	0.09
$\sigma_{residual}$	1.03	1.1	0.62
